# Machine learning-driven risk assessment of severe *Mycoplasma pneumoniae* in children: analysis based on core clinical and immunological features

**DOI:** 10.3389/fmed.2026.1843690

**Published:** 2026-07-21

**Authors:** Duoduo Li, Li Wang, Xiaolu Zhao, Luyang Guo, Yishuai Ren, Xixia Guo, Weihong Lu, Xiangtao Wu, Fenglian Zhu

**Affiliations:** 1 Department of Pediatrics, The First Affiliated Hospital of Henan Medical University, Weihui, Henan, China; 2 Department of Nephrology, The First Affiliated Hospital of Henan Medical University, Weihui, Henan, China

**Keywords:** child, machine learning, *Mycoplasma*, *pneumoniae*, risk assessment

## Abstract

**Objective:**

To construct and validate an interpretable machine learning model for early prediction of severe *Mycoplasma pneumoniae* pneumonia (SMPP) in children.

**Methods:**

Clinical and immunological data of 402 pediatric MPP patients were randomly divided into a training set (70% for model tuning) and a held-out internal test set (30% for final evaluation). We systematically evaluated 113 algorithms based on data collected within 24 h of admission.

**Results:**

The Gradient Boosting Machine (GBM) demonstrated optimal performance, achieving an AUC of 0.805 (95% CI: 0.724–0.886) on the held-out internal test set. The model identified 16 core predictors: dyspnea, C-reactive protein (CRP), total T lymphocytes (CD3+), sputum plug formation (PB), CD3+CD4+CD8− T cells, CD3+CD56+NKT cells, LDH, IL-6, creatine kinase (CK), PCT, ALT, IgA, abnormal coagulation function (D-dimer), CK-MB, erythrocyte sedimentation rate (ESR), and MP-DNA load. SHapley Additive exPlanations (SHAP) analysis revealed that dyspnea, elevated CRP, and decreased T lymphocytes synergistically drive severe illness risk.

**Conclusion:**

The GBM-based model effectively predicts SMPP risk. Pending prospective multi-center validation, we plan to embed this transparent, data-driven tool into electronic medical record (EMR) systems to provide real-time early warning and individualized risk assessment.

## 1 Introduction

*Mycoplasma pneumoniae* pneumonia (MPP) is an important etiological type of community-acquired pneumonia (CAP) in children, with a particularly high proportion among school-aged children (1, 2). Most children have a self-limiting course and a good prognosis, but in recent years, due to factors such as large-scale epidemics, the increase in drug-resistant strains, and reinfection, severe MPP (SMPP) has been on the rise. It can lead to serious pulmonary complications such as necrotizing pneumonia, atelectasis, and pulmonary embolism, and is accompanied by myocardial damage, abnormal liver function, anemia, central nervous system involvement, and multiple organ failure, seriously threatening the life and long-term quality of life of children (2, 3). Previous studies have shown that children with SMPP have a longer duration of fever, higher peak body temperature, and a significantly increased incidence of pulmonary consolidation, pleural effusion, and cardiovascular, hematologic, and coagulation abnormalities compared to non-severe children, often requiring more aggressive glucocorticoid or gamma globulin pulse therapy (4, 5).

In clinical practice, early and accurate identification of high-risk children with a tendency to develop severe illness is key to reducing complications and mortality. However, the early clinical manifestations of SMPP lack specificity and often overlap with those of general MPP or pneumonia caused by other pathogens (3, 6). Traditional risk assessments often rely on single experimental indicators (such as CRP, LDH, IL - 6) or scoring models based on logistic regression. Although these have suggested that inflammatory factors, coagulation indicators, and some imaging features are closely related to the severity of the disease, they are still insufficient in handling high-dimensional, non-linear, and multimodal data (4, 7). At the same time, some existing prediction tools have limitations such as limited sample size, specific age groups, or single centers, and insufficient integration and utilization of immunological features (such as lymphocyte subsets, cytokine profiles, and comprehensive immune inflammation index), making it difficult to fully characterize the evolution of severe illness driven by host immune imbalance (8–10).

In recent years, machine learning (ML) has demonstrated significant advantages in pneumonia severity stratification and complication prediction due to its ability to mine complex non-linear patterns in large-scale, multidimensional clinical data. For SMPP, studies utilizing various algorithms such as LightGBM and CatBoost have achieved AUCs between 0.88 and 0.97, significantly outperforming traditional scoring systems (3, 11). However, the fundamental necessity for such advanced algorithms stems from the complex biological nature of SMPP. The progression to severe illness is driven by a non-linear dynamic imbalance between an intense “pro-inflammatory response” and host “immune exhaustion or suppression” (2, 10). Traditional risk assessments based on logistic regression often rely on the assumption of linearity and fail to capture the intricate interaction patterns inherent in this immune-inflammatory network (4, 7). For instance, a critical clinical “double-hit”—where high C-reactive protein (CRP) levels coincide with a significant decrease in total T-lymphocytes—creates a synergistic risk surge that far exceeds the simple additive effect of these markers, a pattern that linear models are mathematically ill-equipped to identify.

Nevertheless, current research still has several shortcomings: First, most models focus on routine laboratory indicators and lack a systematic integration of specific immunophenotypes—such as T/B cell subset imbalances and cytokine networks (notably IL-6 and IL-18)—that play a core role in the development of SMPP (2, 10). The complex interaction effects between these cellular immune markers and traditional inflammatory indicators remain underexplored in current frameworks. Second, the performance comparison between different algorithms is not systematic enough, and there remains a lack of a unified risk assessment framework based on “core clinical + immune features.” Third, while some models have used interpretable AI technologies like SHapley Additive exPlanations (SHAP) to alleviate the “black box” problem (2, 11), integrated tools for pediatric critical care management, organ damage risk assessment, and treatment decision support are still insufficient (12, 13). This necessitates a more sophisticated algorithmic approach, like the Gradient Boosting Machine (GBM) combined with SHAP, to decode the high-dimensional biological complexity of pediatric SMPP.

Against this backdrop, this study seeks to address the critical clinical uncertainty in identifying which children with *Mycoplasma pneumoniae* pneumonia (MPP) will progress to severe illness during the early stages of admission. Given the non-specific early manifestations of SMPP and the complex interplay of host immune drivers, we aimed to resolve whether an interpretable machine learning framework could accurately decode these patterns by integrating core clinical information (disease course, symptoms, and signs, presence of sputum plugs, and complications) with key immune-related indicators (inflammatory factors, immune cell subsets, and comprehensive inflammatory indexes). By systematically comparing the predictive performance and interpretability of various machine learning algorithms, we sought to construct a transparent and precise risk assessment tool that directly addresses the diagnostic gaps in frontline clinical practice. The ultimate goal is to provide robust evidence-based support for individualized risk stratification, thereby facilitating the development of early intervention strategies and the precise, timely application of immunomodulatory therapy to mitigate the risk of permanent pulmonary damage and improve long-term prognosis in pediatric patients.

## 2 Research methods

### 2.1 Research subjects and data collection

This is a retrospective cohort study. Children diagnosed with MPP and admitted to the Department of Pediatrics of the First Affiliated Hospital of Henan University of Medical Sciences between January 2019 and December 2024 were consecutively included (initial cohort of 2,358 patients). Following the application of various clinical exclusion criteria (totaling 1,956 excluded patients), and to ensure the robust performance of the high-dimensional machine learning models, we implemented a strict “complete case analysis” strategy. Specifically, 699 patients were excluded during the initial screening phase due to missing values in critical laboratory or immunological parameters. While this approach reduces the risk of noise from inaccurate imputations, it results in a final cohort of 402 children with high-quality, verified data for modeling.

#### 2.1.1 Inclusion criteria

(1) Children aged between 28 days and 18 years. (2) Clinical diagnosis (14): Meeting the clinical manifestations of community-acquired pneumonia (such as fever, cough, lung rales) and imaging evidence (chest X-ray or CT showing lung inflammation). (3) Etiological diagnosis (15): Serum *Mycoplasma pneumoniae* antibody (IgM) titer ≥1:160, or a 4-fold or greater increase in antibody titer between acute and convalescent phase serum samples, or detection of *Mycoplasma pneumoniae* DNA in respiratory specimens by polymerase chain reaction. (4) Having complete clinical, laboratory, and immunological examination records that can be used for analysis.

SMPP diagnostic criteria (5): Children were classified as having severe MPP if they met any one of the following core diagnostic criteria (i.e., an “OR” relationship exists among criteria 1 through 8):

1. Deterioration of general condition: Poor mental response, lethargy, or altered consciousness.
2. Significant vital sign abnormalities (Tachycardia AND Tachypnea based on age): under 1 year old: respiratory rate ≥50 breaths/min, heart rate ≥150 beats/min; 1–5 years: respiratory rate ≥40 breaths/min, heart rate ≥140 beats/min; over 5 years: respiratory rate ≥30 breaths/min, heart rate ≥120 beats/min.
3. Hypoxemia: pulse oxygen saturation (SpO₂) ≤ 0.93 when breathing air at rest.
4. Signs of assisted respiration: Presence of groaning, nasal flaring, or three-recession sign (suprasternal notch, supraclavicular notch, intercostal retraction).
5. Severe oxygenation index <250 mmHg.
6. Hypercapnia.
7. Elevated blood urea nitrogen.
8. Hypotension requiring fluid resuscitation.

#### 2.1.2 Exclusion criteria

(1) Co-infection with other pathogens: Confirmed or highly suspected co-infection with bacteria, viruses (such as influenza virus, respiratory syncytial virus), fungi, or tuberculosis. (2) Interference from underlying diseases: Severe primary immunodeficiency, congenital heart disease, hereditary metabolic disease, malignant tumor, autoimmune disease, or other chronic underlying diseases that may significantly affect immune status or course of pneumonia. (3) Interference from pre-admission treatment: Systemic treatment (such as long-term use of immunosuppressants) that may significantly affect the observation indicators of this study was received before this admission. (4) Incomplete data: Key clinical data or laboratory test results are missing, making it impossible to group or analyze. (5) Non-first infection or non-acute phase: Recurrent *Mycoplasma pneumoniae* infection or in the non-acute infection phase.

#### 2.1.3 Information collection

Clinical data of all children admitted to the hospital within 24 h were collected through the hospital’s electronic medical record system.

Demographic characteristics: age, sex;
Clinical manifestations and signs: number of days of illness, number of days of cough, number of days of fever, dyspnea, sputum plug formation (PB), pleural effusion.liquid;
Laboratory indicators: Inflammatory markers: white blood cell count (fully automated hematology analyzer, Beckman Coulter), C-reactive protein, procalcitonin, interleukin-6 (chemiluminescent immunoassay, Beckman Coulter Access 2), erythrocyte sedimentation rate (fully automated dynamic erythrocyte sedimentation rate analyzer, Wuhan DIS); Tissue damage and metabolic indicators: lactate dehydrogenase, aspartate aminotransferase, alanine aminotransferase, creatine kinase, creatine kinase isoenzyme (fully automated biochemical analyzer, Beckman Coulter), D-dimer (fluorescence immunochromatography, Shanghai Aopu Biotechnology), *Mycoplasma pneumoniae* sequence count (real-time quantitative PCR instrument, Thermo Fisher); Immunoglobulin and complement levels (IgG, IgA, IgM, C3, C4) (immunoturbidimetric assay, Thermo Fisher Optilite);

Immune cell subsets: Peripheral blood lymphocyte subsets were detected by flow cytometry, including total T lymphocytes (CD3+), cytotoxic T cells (CD3+CD8+%), helper T cells (CD3+CD4+%), double-negative T cells (CD3+CD4+CD8-%), CD4+/CD8+ratio, B lymphocytes (CD19+%), natural killer cells (CD56+NK%), and natural killer T cells (CD3+CD56+T-NK%) (flow cytometry, BD FACSCanto, FACSAria); ⑤ Treatment and outcome: number of bronchoalveolar lavages, length of hospital stay, number of days of glucocorticoid use, number of days of antibiotic use, whether it is a refractory case, whether intravenous immunoglobulin was used, and the occurrence of cardiovascular, hematologic, urinary, hepatic and other systemic complications and systemic inflammatory response syndrome. Importantly, all variables in this “Treatment and outcome” category were strictly utilized only for baseline clinical comparison and to illustrate disease burden (as shown in Supplementary Table 1). To definitively prevent target leakage, none of these post-admission interventions or outcomes were included in the candidate feature pool for machine learning modeling.

#### 2.1.4 Ethical review and informed consent

This study protocol has been reviewed and approved by the Ethics Committee of the First Affiliated Hospital of Henan University of Medicine (Approval Number: [EC-LW026-058]). Given that this was a retrospective analysis of de-identified and anonymized clinical data, the research posed no more than minimal risk to the participants and did not adversely affect their rights or welfare. Consequently, the Ethics Committee formally approved an exemption from obtaining informed consent, in strict accordance with the Declaration of Helsinki.

### 2.2 Machine learning model construction and selection

The total dataset was randomly divided into a training set and a held-out internal test set in a 7:3 ratio. On the training set, we systematically trained and evaluated 113 different machine learning algorithms and their ensemble models available within the caret and caretEnsemble package frameworks. These 113 algorithms represent distinct base models and their specific combinatorial ensemble variants, rather than simply hyperparameter tuning variants of a single model. To ensure systematic exploration, these algorithms were structured around several core base model families: (1) Tree-based models (e.g., Gradient Boosting Machine, Random Forest, Extreme Gradient Boosting); (2) Generalized Linear Models and Penalized Regressions (e.g., glmBoost, Elastic Network, Lasso, Ridge); (3) Support Vector Machines; and (4) Probabilistic/Discriminant approaches (e.g., Naive Bayes, Linear Discriminant Analysis). Alongside these base algorithms, we generated extensive combinatorial sub-lists of ensemble variants (e.g., glmBoost+GBM, RF+GBM). A comprehensively categorized list of all 113 base models and their specific ensemble variants is provided in Supplementary Table 2. By plotting heatmaps of the AUC values of all models on the training and test sets, we sorted the models in descending order based on their test set AUC, initially selecting the top four performing models for further analysis.

A total of 402 MPP patients were ultimately included. Based on whether they developed severe illness, the children were divided into a non-severe group (*n* = 277) and a severe group (*n* = 125, 31.09%), ensuring a robust event rate for predictive modeling. The cohort was then divided into a training set (*n* = 282) and a held-out internal test set (*n* = 120) in a 70:30 ratio. On the training set, we systematically trained and evaluated 113 different machine learning algorithms and their ensemble models. By plotting heatmaps of the AUC values of all models on the training and test sets, we sorted the models in descending order based on their test set AUC, initially selecting the top four performing models for further analysis. To rigorously avoid data leakage, all subsequent feature selection procedures were executed exclusively on the training set.

### 2.3 Feature selection and determination of the optimal number of features

To construct a concise and efficient clinical prediction model and prevent overfitting, we performed feature importance analysis on four initially selected high-performance models. Using Recursive Feature Elimination (RFE) with 10-fold cross-validation strictly within the bounds of the training set, we evaluated the trend of model AUC values as the number of included features increased. To ensure the model’s generalization ability, the initial 32 candidate variables (which comprised both univariately significant clinical indicators and theoretically critical immunological parameters) were compressed. We selected the inflection point where model performance plateaued, resulting in a core set of 16 high-information features as the optimal number required to construct the final model.

### 2.4 Model interpretability analysis

The SHAP framework is used to perform global and local interpretability analysis on the final determined optimal model (16). For clinical readers, SHAP values serve as a bridge between the complex algorithm and clinical logic: they translate abstract algorithmic weights into intuitive, individualized risk contributions (e.g., calculating exactly how much a specific CRP level increases or decreases a given patient’s probability of severe illness).

Global Interpretation: Draw a SHAP summary plot, which visualizes the overall impact and directional effect of each feature across the entire patient cohort, rank the importance of all included features according to the mean absolute SHAP value of each feature, and identify the core features that contribute the most to the model’s predictions.

Feature effect analysis: Plotting SHAP dependency plots to visualize the non-linear relationship and critical threshold effects between individual feature values and model output (SHAP value), revealing the direction (positive or negative) and pattern of the marginal contribution of the feature to the predicted risk.

Feature interaction: In the SHAP dependency plot, the interaction effect between two important features can be visualized and analyzed by coloring points with the values of another key feature, i.e., how the influence of one feature changes as the value of the other feature changes.

Local interpretation: Select representative samples (high-risk and low-risk), draw SHAP force plots to provide a “patient-specific risk fingerprint.” These plots decompose the model’s overall prediction into the sum of baseline risk and the individual push/pull effects of each clinical feature, acting as a direct, visual bedside decision aid to show exactly why the model made a specific prediction for that specific child.

### 2.5 Statistical analysis

Continuous variables were presented as mean ± standard deviation (SD) or median with interquartile range (IQR), depending on the normality of the distribution. Comparisons between the severe and non-severe groups were performed using the independent samples *t*-test or the Mann–Whitney *U* test, as appropriate. Categorical variables were expressed as frequencies and percentages (*n*, %), and differences between groups were evaluated using the Chi-square test or Fisher’s exact test.

Model performance was comprehensively evaluated using the area under the receiver operating characteristic curve (AUC), sensitivity, specificity, positive predictive value (PPV), and negative predictive value (NPV). Calibration was assessed using the Brier score and calibration plots. Furthermore, to evaluate the clinical applicability of the model beyond mere discriminative accuracy, Decision Curve Analysis (DCA) was conducted. The DCA evaluates clinical utility by calculating the clinical net benefit of the predictive models across various threshold probabilities.

The SHAP (SHapley Additive exPlanations) framework was utilized to perform both global and local interpretability analysis on the optimal GBM model, elucidating the specific contribution and directional impact of each feature (16).

To avoid redundancy in reporting, all statistical analyses and machine learning modeling procedures throughout this study were uniformly conducted using the R software environment (version 4.3.3). A two-sided *p*-value < 0.05 was considered statistically significant for all tests.

Furthermore, to explicitly quantify and address potential selection bias arising from our complete-case analysis strategy, a rigorous sensitivity analysis was conducted. Multiple Imputation by Chained Equations (MICE) was applied to the expanded initial cohort (*n* = 1,101, including patients previously excluded due to missing data). The predictive model was then re-evaluated on this imputed dataset to verify the robustness and generalizability of the original findings.

## 3 Results

### 3.1 Basic characteristics and differences analysis

This study included 402 participants, of whom 277 were non-SMPP (68.91%), and 125 were SMPP (31.09%) (Supplementary Figure 1). Statistically significant differences were found between the two groups in baseline parameters including CRP, PCT, LDH, Il6, D2, ALT, AST, CK, immunoglobulin A, CD3+CD4+CD8−, length of hospital stay, days of hormone use, days of antibiotic use, dyspnea, sputum plug formation (PB), pleural effusion, cardiovascular function, blood (anemia), urinary function, systemic inflammatory response syndrome, coagulation function, and intravenous immunoglobulin (IVIG) use (*p* < 0.05). Variables reflecting post-admission outcomes or interventions have been excluded from the baseline comparisons. Detailed demographic and clinical baseline characteristics of the study population are summarized in Supplementary Table 1. It is important to reiterate that all post-admission intervention and outcome variables were evaluated solely to illustrate the clinical burden of the disease and were strictly excluded from the candidate feature pool during predictive modeling.

### 3.2 The optimal model for predicting the risk of severe childhood pneumonia (MPP) was selected using machine learning algorithms

**Figure 1:**
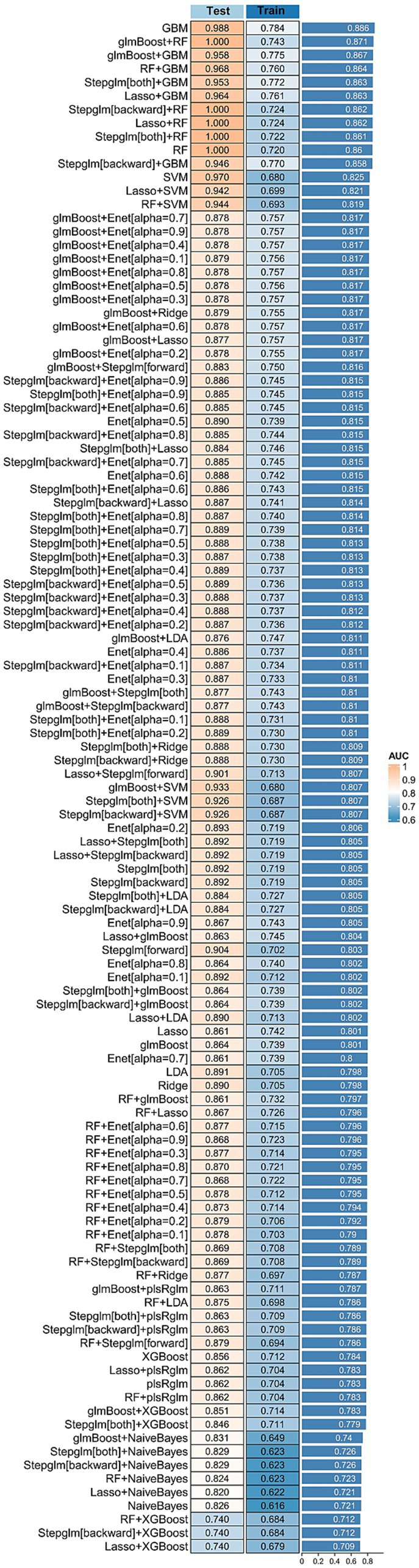
Performance comparison of different machine learning models in predicting the risk of severe *Mycoplasma pneumoniae* pneumonia. The horizontal axis represents the area under the curve (AUC). Models are sorted in descending order of AUC values on the test set (orange bars). Blue bars represent AUC values on the training set. The third, rightmost column explicitly displays the mean value of the training and test set AUCs for each corresponding model. GBM, Gradient Boosting Machine; RF, Random Forest; SVM, Support Vector Machine; Enet, Elastic Network; Lasso, Minimum Absolute Shrinkage and Selection Operator; Ridge, Ridge Regression; glmBoost, Generalized Linear Model Boosting; Stepglm, Stepwise Generalized Linear Model; plsRglm, Partial Least Squares Generalized Linear Model; XGBoost, Extreme Gradient Boosting; NaiveBayes, Naive Bayes; LDA, Linear Discriminant Analysis.

This study first aimed to screen the optimal model for predicting the risk of severe childhood pneumonia (MPP) from a large number of machine learning algorithms. We systematically trained and evaluated 113 different machine learning models and model combinations. Figure 1 shows the area under the receiver operating characteristic (AUC) curves of all models on the training and held-out internal test sets, and is a heatmap ranked in strict descending order of the models’ performance (AUC) on the held-out internal test set. Figure 1 clearly shows that the model performance exhibits a significant stratification. The models at the top of the leaderboard performed exceptionally well, with the Gradient Boosting Machine (GBM) model achieving the most reliable predictive performance on the held-out internal test set (AUC = 0.805, 95% CI: 0.724–0.886), while its training set AUC of 0.988 served only as an internal reference for model fitting during the training phase. Following closely behind were high-performing models, including glmBoost+RF (test set AUC: 0.743), glmBoost+GBM (test set AUC: 0.775), and RF+GBM (test set AUC: 0.760). These four “top performers” all had test set AUCs above 0.75 and were initially identified as candidate models with the best predictive performance, proceeding to further in-depth analysis. Also reveals a key issue: some models exhibit significant performance differences between the training and test sets, indicating obvious overfitting. The most typical example is the Random Forest (RF) model, which achieves a perfect AUC (1.000) on the training set, but its performance on the test set (AUC: 0.720) is significantly lower than models like GBM. Similarly, combined models incorporating RF, such as Lasso+RF and Stepglm [both]+RF, show a similar pattern—extremely high training set AUC and relatively mediocre test set AUC. Notably, the pure GBM model, with its highest test set discriminative power, demonstrates its core potential as a final prediction tool.

### 3.3 Optimal predictive performance of the model under different numbers of clinical features

**Figure 2:**
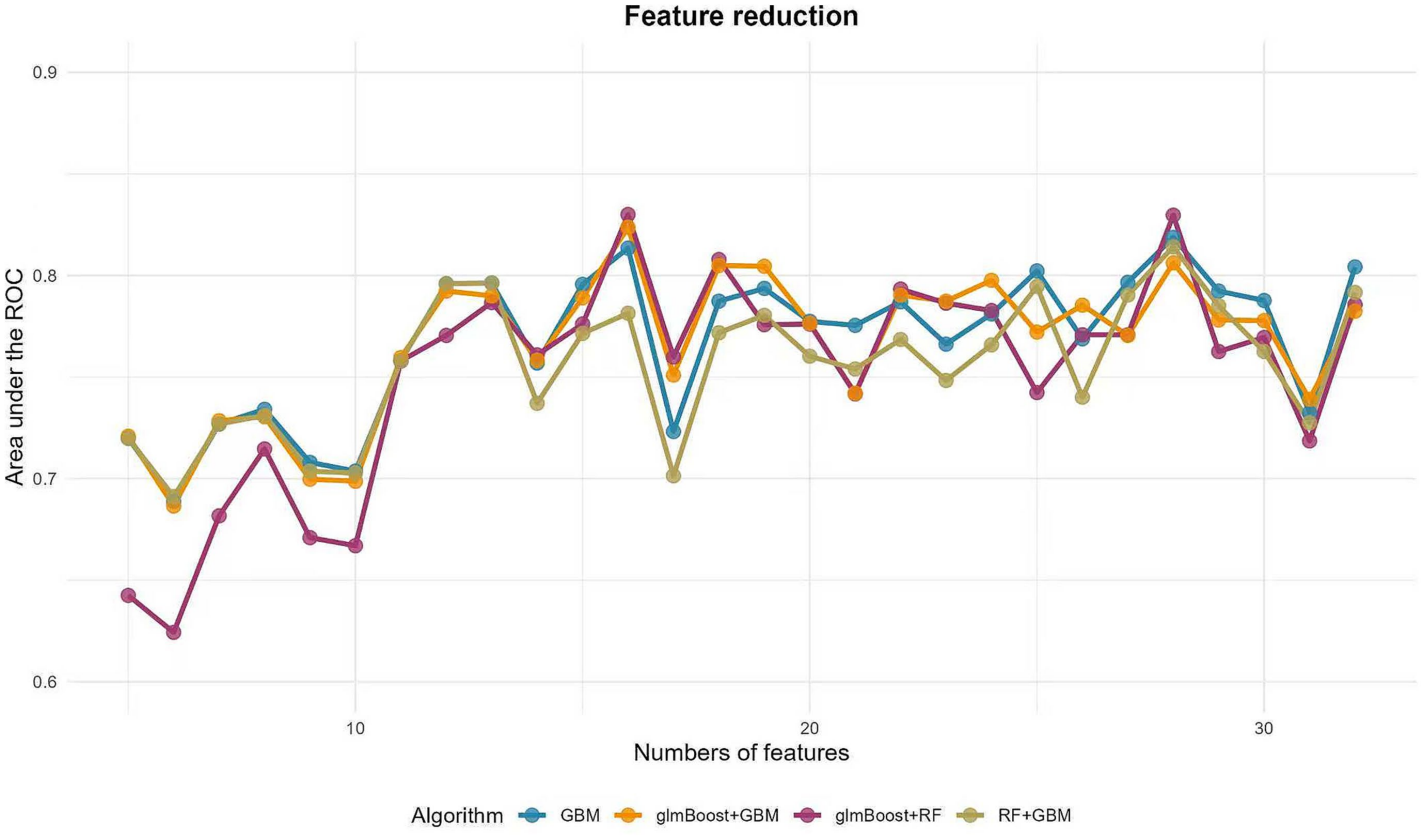
Illustrates the model prediction performance (measured by the area under the ROC curve, AUC) of different machine learning algorithms (GBM, glmBoost+GBM, glmBoost+RF, and RF+GBM) with varying numbers of features. The horizontal axis represents the number of features included in the model (approximately 1–30), and the vertical axis represents the AUC value (0.6–0.9). The different colored lines represent the four algorithms: GBM (blue), glmBoost+GBM (orange), glmBoost+RF (purple), and RF+GBM (green).

After selecting four high-performance models (GBM, glmBoost+GBM, glmBoost+RF, and RF+GBM) through heatmaps, we conducted a systematic feature dimensionality reduction analysis to determine the optimal number of features required to build the predictive model. Figure 2 shows the performance (AUC) trends of these four models under different numbers of features (from approximately 5 to 32). As shown, the AUC values of all models exhibit a typical pattern of rapid increase, plateau, and eventual stabilization with the increase in the number of features included. Specifically: Initial stage (feature count < 10): When the number of features is small, the AUC values of all four models are relatively low (approximately 0.7–0.75), indicating that the limited information is insufficient to fully characterize the complex patterns of severe illness risk. Rapid improvement and plateau (feature count approximately 10–16): As the number of features increases to 10–16, the AUC values of all models rapidly increase to near or reach their peak, forming a clear performance plateau. Of particular note is that when the number of features reaches 16, the AUC values of all four models simultaneously reach or are very close to their respective maximum values across the entire interval. For example, the GBM model has an AUC of approximately 0.78 with 16 features, which is comparable to or even better than its performance when using all 32 features. Saturation and fluctuation stage (number of features > 16): When the number of features continues to increase to more than 20, the model’s AUC value no longer increases significantly, but instead shows a slight fluctuation or a slight downward trend. This indicates that after exceeding 16 features, the additional features may not provide new effective information, or may introduce redundancy and noise, leading to a failure to further improve model performance or even the risk of overfitting. Therefore, to construct an efficient, robust, and clinically applicable model for predicting the risk of severe pneumonia in children, the model should be trained based on 16 core clinical immune features.

### 3.4 ROC curve of the performance of the childhood pneumonia severe illness risk prediction model

**Figure 3:**
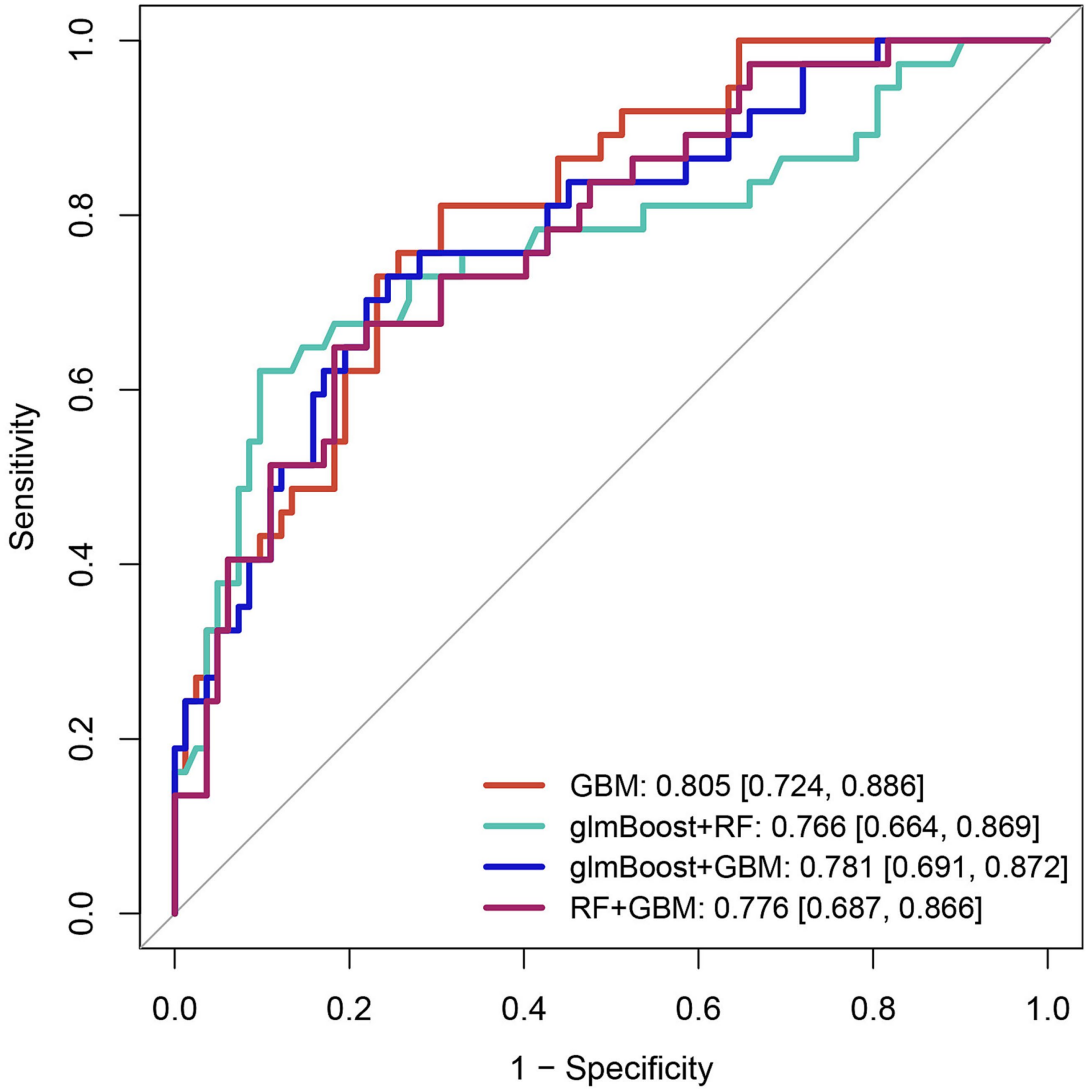
ROC curve comparison of the performance of the childhood pneumonia severe illness risk prediction model. This figure compares the performance of four machine learning models in predicting the risk of severe pneumonia in children. The horizontal axis (1-Specificity) represents the false positive rate, and the vertical axis (Sensitivity) represents the true positive rate (sensitivity). The four colored curves in the figure correspond to four different combinations of machine learning models: glmBoost+RF, GBM, RF+GBM, and glmBoost+GBM. The gray dashed line (*y* = *x*) in the figure represents the baseline where the model’s predictive ability equals random guessing (AUC = 0.5). The area under the ROC curve (AUC) for each model is labeled in the legend in the lower right corner of the figure. The closer the ROC curve is to the upper left corner, the better the overall discriminative performance of the model.

Figure 3 clearly shows the positions of the ROC curves and their AUC values for the four models on the held-out internal test set. The Gradient Boosting Machine (GBM) model has the highest AUC value of 0.805, and its ROC curve is closest to the top left corner, which intuitively confirms its position as the best-performing model in the heatmap analysis. The AUC values of the other three models are: glmBoost+GBM (0.781), RF+GBM (0.776), and glmBoost+RF (0.766). Pairwise comparisons using DeLong’s test indicated no statistically significant differences in AUC among these top four models (all *p* > 0.05). Therefore, through direct comparison of the ROC curves and AUC values, and considering the principle of model parsimony (avoiding overly complex ensembles) alongside its strong native compatibility with tree-based interpretability frameworks, the GBM model (AUC = 0.805) was ultimately identified as the optimal algorithm for constructing the prediction tool in this study. At the optimal probability threshold of 0.66, the GBM model demonstrated a Sensitivity of 58.3%, Specificity of 94.0%, PPV of 80.8%, and NPV of 84.0%. Furthermore, the model exhibited good calibration on the held-out internal test set, with a Brier score of 0.198, a calibration intercept of −1.047, and a calibration slope of 1.311 (Supplementary Figure 2).

### 3.5 SHAP (force plot) interprets the prediction of severe illness risk in a single sample

**Figure 4:**
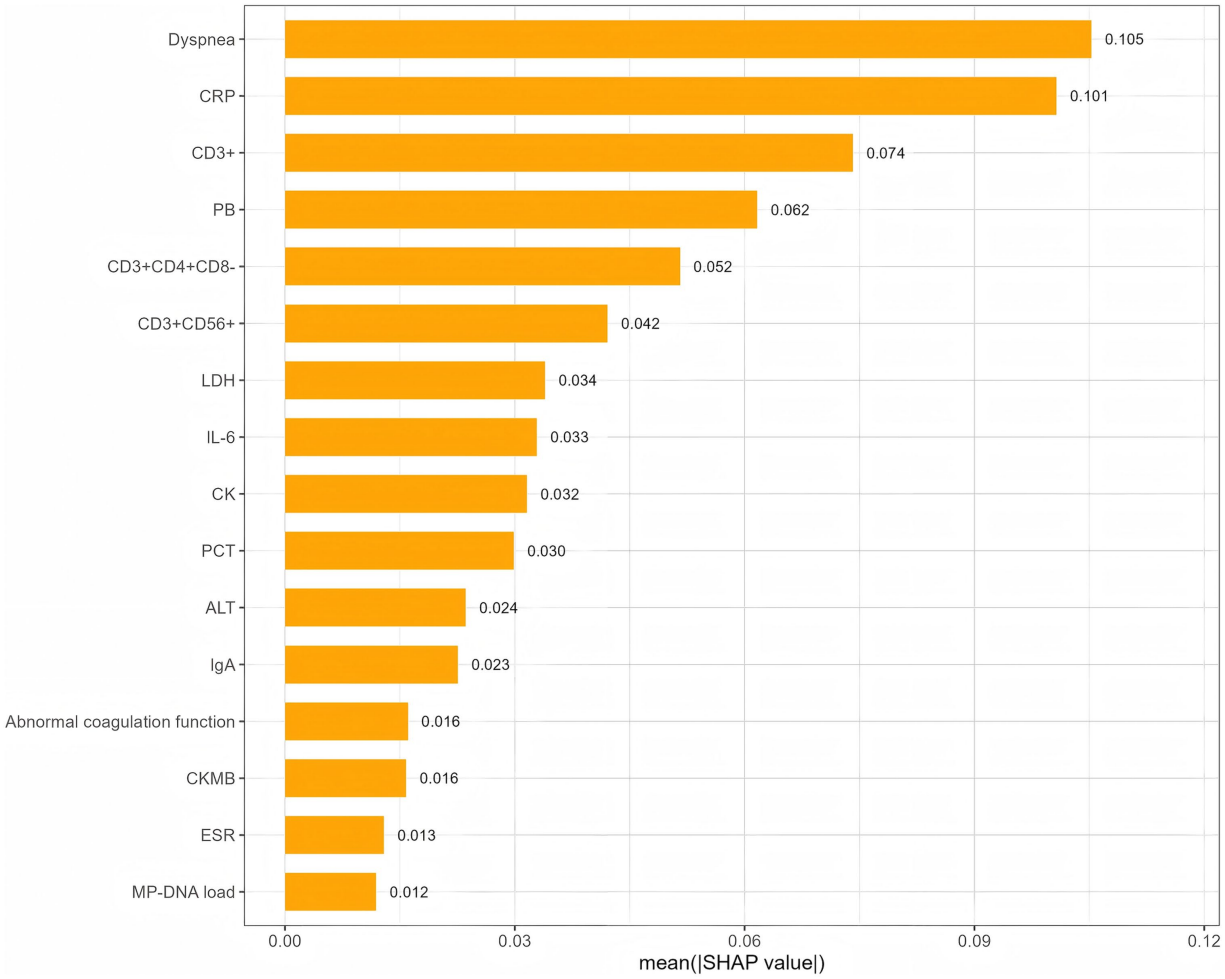
Global feature importance ranking based on SHAP values. This bar chart quantifies the overall contribution of the 16 core clinical and laboratory indicators to the GBM model’s predictions. The horizontal axis represents the mean absolute SHAP value (mean(|SHAP value|)), measuring the average impact of each feature on the model’s output across the entire cohort. Longer bars (e.g., Dyspnea and CRP) indicate a greater overall importance in driving the prediction of severe illness risk.

As shown in Figure 4, the overall contribution of each input feature to the GBM model’s prediction results was quantified based on the SHAP value. The importance of a feature is measured by its mean absolute SHAP value (mean(|SHAP value|)), with a higher value indicating a greater overall impact on the model’s prediction results. This ranking prioritizes the importance of 16 core predictors, providing a data-driven basis for prioritizing key clinical indicators. As shown in the figure, among all features included in the analysis, “dyspnea” is the most important predictor, with a mean absolute SHAP value of 0.105. This is followed by “C-reactive protein (CRP)” with an importance of 0.101. The third most important feature is “total T lymphocytes (CD3+)” with an importance of 0.074. Features of intermediate importance include (in descending order of importance): sputum plug formation (PB) (0.062), double negative T cells (CD3+CD4+CD8−%) (0.052), natural killer T cells (CD3+CD56+T-NK%) (0.042), lactate dehydrogenase (LDH) (0.034), interleukin-6 (IL-6) (0.033), creatine kinase (CK) (0.032), procalcitonin (PCT) (0.030), alanine aminotransferase (ALT) (0.024), immunoglobulin A (IgA) (0.023), coagulation function (D-dimer) (0.016), creatine kinase isoenzyme (CK-MB) (0.016), and erythrocyte sedimentation rate (ESR) (0.013), and MP-DNA load (0.011). The results showed that clinical signs (dyspnea), systemic inflammatory response markers (CRP), and cellular immune status indicators (total T lymphocytes) were the core criteria for the model to assess the risk of severe illness, and their importance was significantly higher than that of other laboratory indicators. This provides clear, visual evidence for understanding the model’s decision-making logic and identifying key clinical risk factors.

### 3.6 Feature effect and non-linear association based on SHAP summary plot

**Figure 5:**
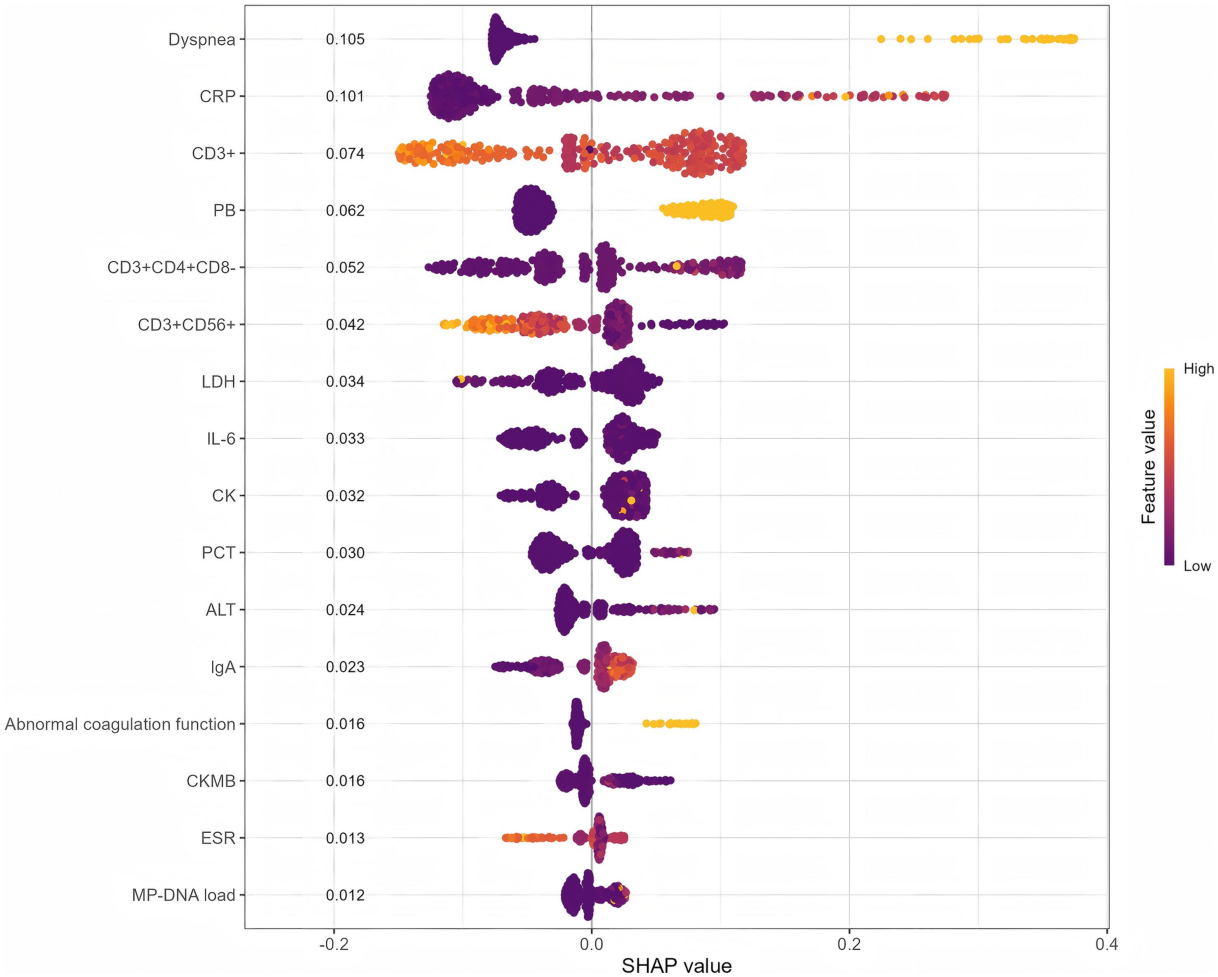
SHAP summary dot plot illustrating feature effects and non-linear associations. This plot visualizes the directional impact of each feature on the individual prediction of severe pneumonia risk. The vertical axis lists the features in descending order of global importance. Each dot represents a single patient. The horizontal axis indicates the corresponding SHAP value; positive SHAP values (points to the right of the vertical zero line) drive the prediction towards severe illness, while negative values push it towards non-severe illness. The color gradient reflects the actual value of the feature for that specific patient (yellow indicates high values, purple indicates low values).

While the previous bar chart establishes global importance, the SHAP summary dot plot (Figure 5) further elucidates the directional effects and non-linear relationships of these core features on risk prediction. In this plot, each dot represents a single patient, colored by the actual value of the feature (yellow for high, purple for low), with its horizontal position indicating the corresponding SHAP value (impact on model output).

The graph clearly reveals how feature variations drive the risk of severe illness. For instance, the binary feature “dyspnea” demonstrates a distinct separation: the presence of dyspnea consistently maps to positive SHAP regions (>0), acting as a strong risk driver. For continuous variables like “CRP,” the scatter plot shows a clear rightward trend, confirming that higher CRP concentrations (yellower dots) correspond to larger positive SHAP values, thus escalating the predicted risk. Conversely, ‘total T lymphocytes’ exhibit a complex distribution where low values (purple dots) are strongly associated with positive SHAP values, highlighting non-linear associations and potential threshold effects that traditional linear models often fail to capture. Most other lower-ranking features have points concentrated near 0, indicating a minimal marginal contribution to individual predictions.

### 3.7 Visualize the interaction effects between key clinical feature pairs

**Figure 6:**
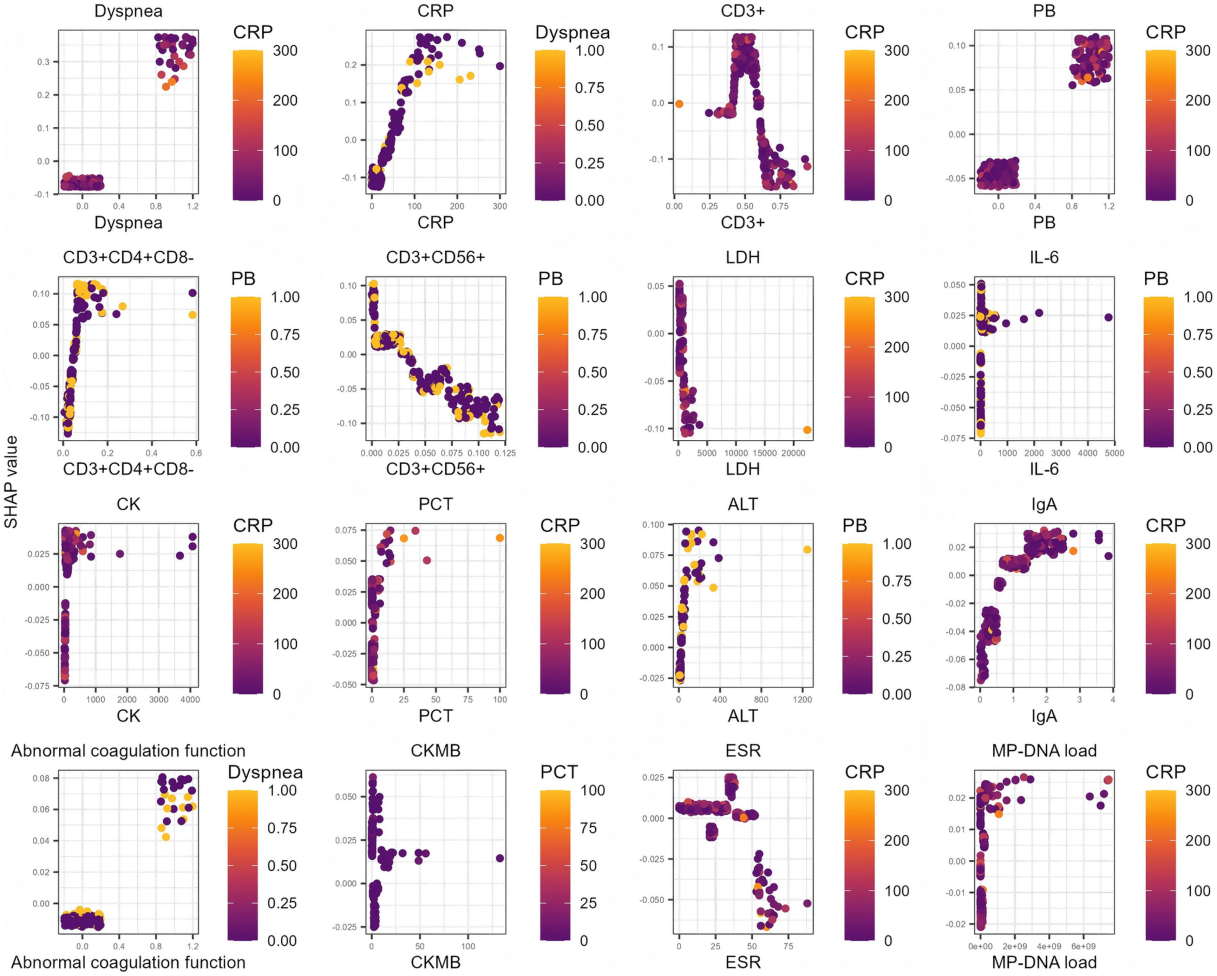
SHAP dependency plots for various clinical and laboratory indicators. This set of figures quantifies and visualizes the independent contributions of 16 clinical/laboratory indicators [such as dyspnea, C-reactive protein, total T lymphocytes, and sputum plug formation (PB)] to the prediction of severe illness risk, as well as their interactions with key features [such as C-reactive protein, sputum plug formation (PB), and procalcitonin]. The horizontal axis represents the value of the feature itself, and the vertical axis represents the SHAP value (positive contributions increase the risk of severe illness, while negative contributions decrease the risk). Color coding associates the value of another key feature (yellow represents a high value of the key feature, and purple represents a low value).

By utilizing the SHAP dependency plots (Figure 6), we analyzed the independent contributions of various clinical and laboratory indicators to the prediction of severe illness risk, and visualized the interaction effects between key feature pairs (i.e., how the predictive impact of one feature changes with the value of another). The results showed that: Dyspnea: its SHAP value increased with its own value, and the high-value area (value ≈ 1.2) was colored orange (corresponding to high C-reactive protein values), indicating that the positive impact of increased dyspnea accompanied by elevated CRP was more significant on the risk of severe illness. C-reactive protein (CRP) SHAP values increased rapidly at first and then stabilized. In the low CRP range (<100), the positive interaction with dyspnea (color dimension) enhanced the risk, while in the high CRP range (>200), the negative interaction with total T lymphocytes (color association in some subplots) partially offset the risk, demonstrating its “double-edged sword” effect. The SHAP value of total T lymphocytes decreased with increasing levels, and the correlation with the color of the dots in the high CRP area showed that low lymphocyte counts accompanied by high CRP had a more significant positive contribution to the risk of severe illness. The SHAP value of sputum plug formation (PB) increased with increasing levels, and the correlation with the color of the dots in the high CRP area showed that significant sputum plug formation (PB), accompanied by high CRP, had a more significant positive contribution to the risk of severe illness. The trends in SHAP values of other characteristics (such as CD3CD56TNK cells, lactate dehydrogenase, interleukin-6) and the interactive characteristics of color coding (such as correlations with CRP and sputum plug formation (PB)) further revealed the independent and synergistic effects of immune function, metabolic indicators, and coagulation function on the risk of severe illness. These dependency graphs not only quantify the marginal contribution of a single feature to risk, but also visualize the interaction between features through the color dimension (associating with the value of another key feature). This provides an intuitive basis for understanding the complex logic of model decision-making (such as combined risk patterns like “dyspnea + high CRP” and “low lymphocytes + high CRP”), and also provides a multi-dimensional reference for the accurate identification of high-risk phenotypes in clinical practice.

### 3.8 Single-sample prediction interpretation based on SHAP force plot

**Figure 7:**
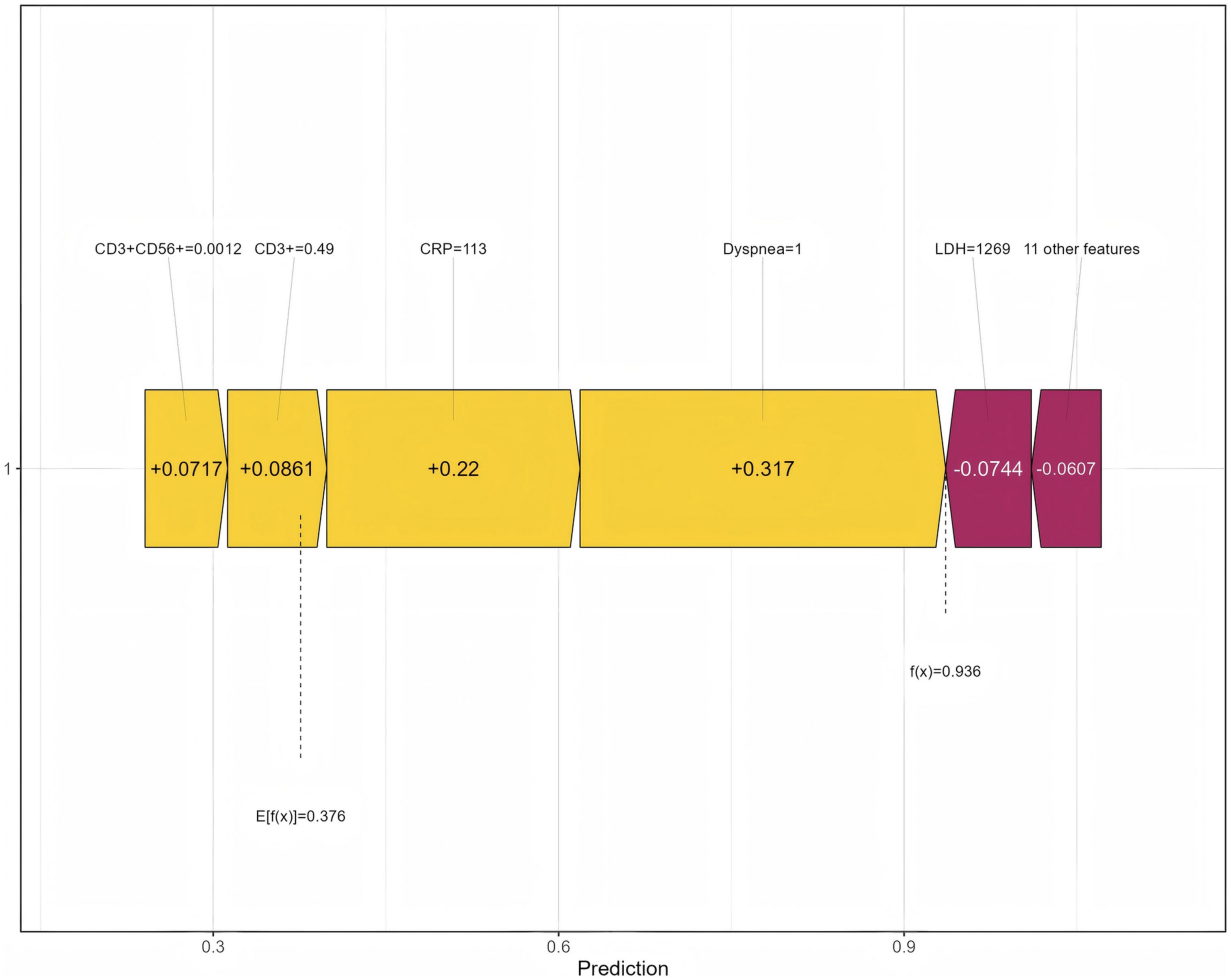
Individual prediction explanation. The SHAP plot of the 10th sample is used to explain the model’s prediction of the “Treatment category probability” for that sample. The horizontal axis represents the prediction value, and the vertical axis shows the SHAP values of each feature and the “11 other features” (the contribution of each feature to the prediction result; positive values increase the prediction result, and negative values decrease it). The figure marks the values of key features and their corresponding SHAP values, and also shows the predicted value of this sample *f*(*x*) = 0.936 and the average predicted value of all samples *E*[*f*(*x*)] = 0.376.

By utilizing the SHAP force plot (Figure 7), we achieved local interpretability of the model’s decision-making by analyzing a high-risk child (predicted probability *f*(*x*) = 0.936) as an example. It decomposes the model’s prediction result into the sum of contributions of each feature: the baseline value (*E*[*f*(*x*)] = 0.376) is the starting point of the model’s average prediction. This figure is a SHAP force plot used to explain the decision-making process of the machine learning model in predicting the risk of severe illness for a single child. The dashed line on the left and the value *E*[*f*(*x*)] = 0.376 represent the model’s average risk prediction value for all patients, i.e., the baseline of the prediction. The solid line on the right and the value *f*(*x*) = 0.936 represent the model’s final prediction result for this specific child, showing that their risk of severe illness is as high as 93.6%. Specifically, the predicted outcome for this child was primarily driven by two indicators: dyspnea = 1 (contribution +0.317) and CRP = 113 mg/L (reference range: 0–8.0 mg/L) (contribution +0.22), which significantly improved the risk prediction value. Secondly, total T lymphocytes (CD3+) = 0.49 (49%)(reference range: 50–84%) (contribution +0.0861) and CD3+CD56+NKT cells = 0.0012 (0.12%) (reference range: 1–5%) (contribution +0.0717) also contributed to the increased risk. Meanwhile, LDH = 1,269 U/L (reference range: 120–250 U/L) (contribution −0.0744) and 11 other features (total contribution −0.0607) had a certain inhibitory effect, but were insufficient to offset the positive driving effect of the former. Overall, this study visually demonstrates that “dyspnea” and “CRP” are the core criteria for the model to determine that this child was severely ill, while also revealing the specific influence of immune indicators in this sample, thus verifying the interpretability of the model’s decision. To further illustrate real-time bedside use across different clinical scenarios, we analyzed an intermediate-risk and a low-risk case based on the model’s SHAP logic. For the intermediate-risk child, the model evaluated a complex presentation where CRP was elevated at 70.0 mg/L (acting as a positive driver for severity), but normal total T lymphocyte counts (CD3+ = 52.8%) and the absence of dyspnea effectively pulled the overall risk back towards the baseline. This counterbalancing effect suggests a need for close clinical observation rather than immediate aggressive intervention. Conversely, in the low-risk case, the synergistic protective effects of normal inflammatory markers (CRP = 2.0 mg/L) and intact immune subsets (total T lymphocytes = 62.43%), alongside the absence of respiratory distress, overwhelmingly dominated the prediction, confidently supporting standard care. (Supplementary Figure 3).

### 3.9 Clinical utility and sensitivity analysis

**Figure 8:**
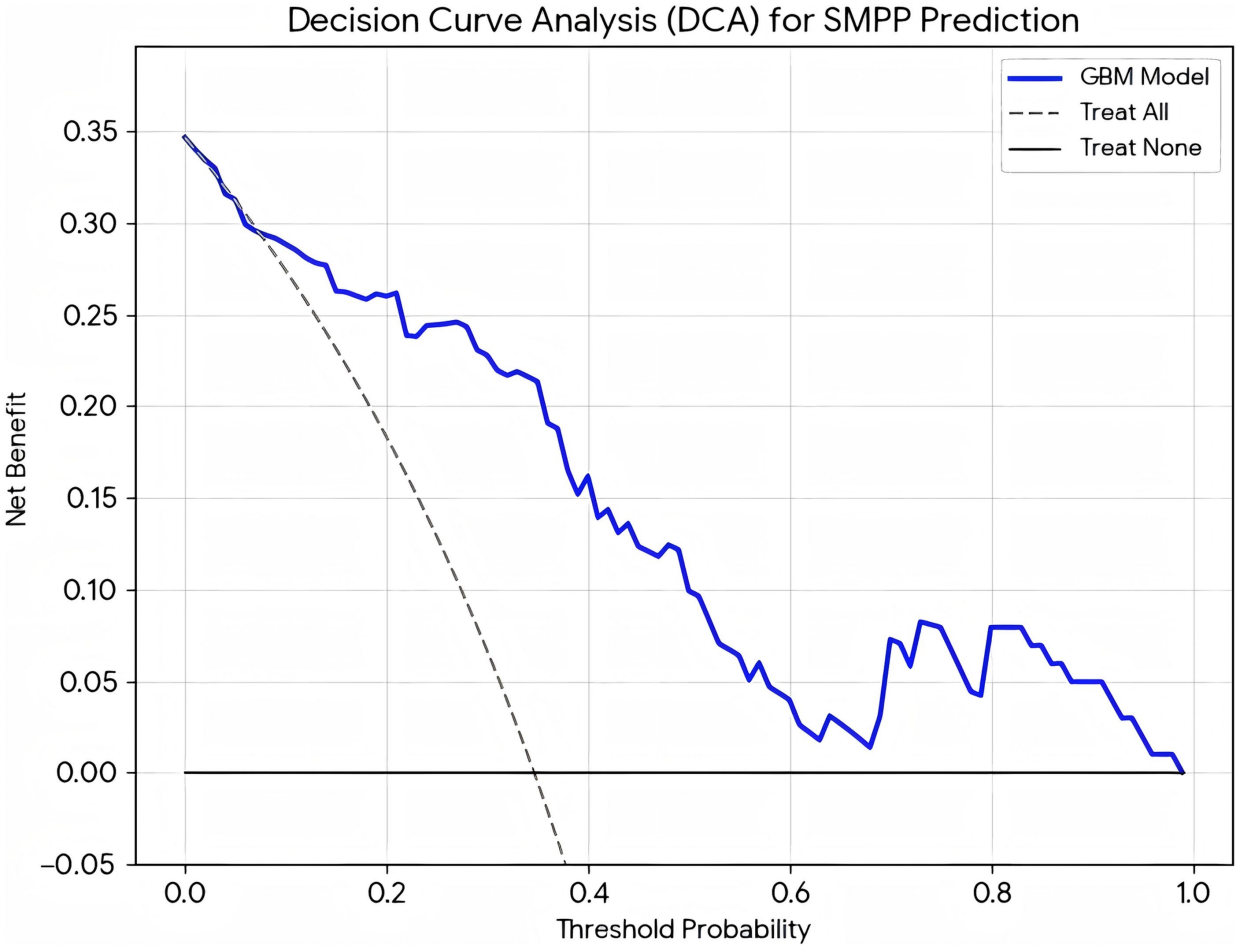
Decision Curve Analysis (DCA) of the GBM model. The blue solid line represents the net benefit of the GBM model across different threshold probabilities. The gray dashed line represents the ‘treat-all’ strategy, and the black horizontal line represents the ‘treat-none’ strategy. The model demonstrates a higher net benefit within the threshold range of 0.1–0.9, indicating its strong potential for clinical decision support.

To evaluate the clinical applicability of the GBM model beyond mere discriminative accuracy, we conducted a Decision Curve Analysis (DCA) (Figure 8). The DCA curve plots the clinical net benefit against a range of threshold probabilities. For thresholds between approximately 0.1 and 0.9, the GBM model demonstrated a higher clinical net benefit compared to both the “treat-all” and “treat-none” strategies. This indicates that using the model to guide early interventions—such as intensified monitoring or targeted immunomodulatory therapy—results in a superior balance of risk and benefit compared to conventional uniform treatment approaches, thereby facilitating precise clinical decision-making. The GBM model, when retrained and evaluated using 10-fold cross-validation on this imputed dataset, demonstrated robust predictive performance with a mean AUC of 0.887 (95% CI: 0.865–0.909). This consistency confirms that the model’s predictive capability and the identified core immunological signatures remain reliable and generalizable across the broader clinical population, including patients presenting with missing baseline variables. To rigorously assess the potential selection bias introduced by excluding patients with missing laboratory or immunological data, we performed a sensitivity analysis on an expanded cohort of 1,101 patients using MICE. The GBM model, when applied to this comprehensively imputed dataset, demonstrated highly consistent predictive performance compared to our primary complete-case cohort. The stability of the AUC and key feature rankings across both cohorts confirmed that the exclusion of incomplete cases did not introduce significant selection bias, thereby validating the robustness and generalizability of our proposed model.

### 3.10 Subgroup analysis and model fairness

To evaluate the robustness of the GBM model across different patient demographics, we performed subgroup analyses on the held-out internal test set (detailed results are provided in Supplementary Table 3). The model demonstrated good discriminative power in children under 5 years (AUC = 0.833) and acceptable predictive performance in children aged 5 years and older (AUC = 0.776). Similarly, the model showed stable performance across sexes, with an AUC of 0.761 for males and 0.791 for females. These findings, summarized in Supplementary Table 2, suggest that while the core clinical and immunological signatures identified are shared drivers of disease severity, clinical application should be age-stratified. For children under 5 years, the model serves as a robust primary risk stratification tool to detect early deterioration. For school-aged children (≥5 years), given their typically stronger immune-inflammatory responses, the model’s predictions should be integrated with close bedside monitoring of inflammatory markers to guide timely interventions.

## 4 Discussion

This study aims to address the clinical challenge of early identification of severe *Mycoplasma pneumoniae* pneumonia (MPP) risk in children using advanced machine learning techniques. Through systematic screening of 113 machine learning algorithms and combinations, we successfully constructed a high-performance predictive model based on Gradient Boosting Machine (GBM), achieving an AUC of 0.805 on the held-out internal test set. More importantly, this study deeply integrates the SHAP interpretability analysis framework, not only optimizing the core clinical feature set but also revealing the complex interactions between key risk factors, providing a transparent and reliable basis for the clinical translation of the model.

Baseline statistical analysis first confirmed the systemic involvement characteristic of severe MPP. Our data showed that critically ill children had a significantly higher incidence of complications in the cardiovascular, hematologic (anemia), coagulation disorders, and systemic inflammatory response syndrome (SIRS) than non-critically ill children. This indicates that severe MPP is not limited to pulmonary infection, but is often accompanied by a strong systemic inflammatory response and multi-system dysfunction (6, 17). In addition, the proportion of critically ill patients receiving intravenous immunoglobulin pulse therapy was significantly higher, which also indirectly reflects the clinical tendency to judge and intervene in the more severe immune inflammatory response in critically ill children (6, 18, 19).

This study systematically screened 113 algorithms and finally selected GBM, which achieved an AUC of 0.805 on the held-out internal test set. While some previous machine learning models for SMPP have reported higher AUCs (ranging from 0.88 to 0.97) (3, 18), these studies often relied heavily on routine laboratory indicators. We acknowledge that the primary novelty of our present model does not rest on maximizing the headline discriminative AUC. Instead, its distinct value lies in its enhanced clinical utility and explanatory depth. By systematically integrating a comprehensive immunological panel (notably T-lymphocyte subsets) with advanced interpretable AI (SHAP), our model moves beyond “black-box” predictions. It provides actionable, mechanistic insights—such as quantifying the ‘double-hit’ synergistic risk of intense inflammation and immune exhaustion—and demonstrates substantial clinical net benefit across a wide range of threshold probabilities via Decision Curve Analysis (DCA) (3, 19). This result shows that although relying solely on a single logistic regression/nomogram can achieve a good level (AUC of about 0.805), gradient boosting algorithms have a significant advantage in characterizing complex patterns (7, 20). GBM outperformed RF in this study, which is consistent with the conclusion in other pneumonia/MPP studies that “gradient boosting algorithms (LightGBM/XGBoost/CatBoost) are often the best models” (3, 19, 21). While the DCA demonstrated a consistent net clinical benefit across a broad range of threshold probabilities, a minor fluctuation (a dip) was observed near the 0.65 threshold. This is likely a statistical artifact driven by the relatively small sample size of patients occupying this specific high-predicted-probability bin in our current cohort, which temporarily introduces noise into the net benefit calculation at that stringent threshold. Nevertheless, the overall trajectory of the DCA curve confirms the robust clinical utility of the integrated model.

In this study, the AUC of the RF training set was 1.000 while the AUC of the test set was 0.720, which typically reflects the tendency of highly complex tree sets to overfit when the sample size is limited and the features are numerous (2, 22). Held-out internal test sets or prospective cohorts must be used to evaluate the model, rather than simply reporting training/internal validation results (3, 18, 20). This study used held-out internal test sets for validation, which better demonstrates the model’s generalization ability compared to studies that only perform internal cross-validation or single-center internal validation (3, 22).

Multiple clinical prediction studies have shown that performance tends to stabilize after the number of features increases to a certain range, and further increases have limited benefits. In Acute Kidney Injury (AKI) and in-hospital mortality prediction, after compressing hundreds of EMR features to 35 and 54 through multi-step feature selection, the AUC did not decrease significantly, and the stability and similarity were the best (22). In cardiovascular, stroke, and other risk prediction, by using various feature selection methods to compress the initial 30–300 variables into about 5–21 core features, the AUC only decreased slightly or remained basically unchanged (23–25). The results of this study show that the AUC rises rapidly and enters a plateau period when there are 10–16 features, while >16 features introduce noise and cause fluctuations, which is highly consistent with the above-mentioned rule that “a small number of high-information features are sufficient to support high performance” (23, 26).

Using SHAP for feature ranking and dependency graph analysis aligns with the mainstream practice of “using SHAP to alleviate the black box” in SMPP and severe pneumonia models in recent years (3, 18). Similar to the synergistic effect of “dyspnea + CRP double hit” in this study, other studies have also suggested that when high inflammatory markers (CRP, S100A8/A9, IL - 6, LDH) are superimposed with imaging/symptom severity, the risk of severe illness or organ damage increases significantly (6, 22, 27). SHAP can not only explain the importance of features at the population level, but also be used for individual decision visualization to support bedside shared decision-making (2, 11). The SHAP summary plot shows that “dyspnea” and “C-reactive protein (CRP)” are the two features that contribute the most to the model prediction, which strongly confirms from the perspective of data science the rationality of using respiratory distress signs and the intensity of systemic inflammatory response as the primary basis for judging the severity of the disease in clinical practice (28). The fact that “low total T lymphocytes” ranked third is strongly associated with MPP disease (9). The coexistence of low immune function (low CD3+) and strong inflammatory response (high CRP) is an independent predictor of an increased risk of severe illness. In the initial univariate analysis (Supplementary Table 1), there was no statistically significant difference in total T lymphocytes between the severe and non-severe groups (*p* = 0.110). However, in the machine learning model, it was identified as the third most important predictor. This highlights that machine learning algorithms can capture the complex nonlinear relationships and interaction effects between variables, thereby discovering those variables that do not have significant effects on the outcome when they are alone, but have an important impact on the outcome under the combined effect of multiple factors (29). In this study, dyspnea, CRP, and T lymphocytes together constitute the cornerstone of the model judgment - that is, the combination of clinical signs of critical illness and strong systemic inflammatory response and host cell immune status is the most direct signal for identifying severe illness. Other indicators (such as LDH, IL-6, D-dimer, etc.) contribute relatively little, but they enrich the model’s judgment criteria from different perspectives (tissue damage, cytokine storm, coagulation disorders) and may play a synergistic role in the interaction with core features. This corroborates the findings of other studies that identified “sputum plugs, persistent high fever, D-dimer, LDH, NLR, CRP, and immune cell subsets” as key factors for predicting severe illness (6, 18, 20). This quantitative “double-hit” phenomenon accurately depicts the process of coordinated deterioration of the condition in clinical practice, suggesting that clinicians should pay close attention to and intervene early in children who simultaneously exhibit severe respiratory symptoms and high inflammatory markers. Therefore, the 16 core indicators selected in this study constitute a multi-dimensional, hierarchical, and interactive set of clinical-immunological features. It transcends the traditional approach of relying on a single or a few biomarkers and constructs a comprehensive assessment framework. By integrating this information, the GBM model achieves an accurate prediction of the risk of severe MPP in children. This result provides a usable predictive tool and deepens the understanding of severe MPP clinical phenotypes, suggesting that the condition is characterized by both intense inflammatory damage and imbalance/deficiency of the host immune response (9).

In this study, the SHAP framework transforms the GBM model from a “black box” into a transparent clinical assistant. While global importance plots identify dyspnea, CRP, and T-lymphocyte count as the primary drivers across the cohort, the true strength of SHAP lies in its ability to provide “local” or individualized explanations.

As illustrated in the individual prediction analysis (Figure 7), SHAP quantifies how each clinical and immunological feature “pushes” or “pulls” the final risk probability for a specific patient. For instance, in a child who presents with only moderate fever but shows a significantly high predicted risk (e.g., *f*(*x*) = 0.936), SHAP analysis can reveal that the risk is predominantly driven by a profound depletion of total T-lymphocytes (CD3+) and elevated IL-6, even if traditional markers like CRP are not yet at critical levels. This individualized “risk fingerprint” allows clinicians to look beyond simple threshold-based diagnoses and understand the specific biological drivers of severity in each case.

Furthermore, this level of explainability addresses the “trust gap” in medical artificial intelligence. By visualizing the evidence-based logic behind a prediction, clinicians can cross-reference the model’s output with their own bedside observations. This is particularly valuable for identifying “high-risk, low-inflammation” phenotypes where immune exhaustion may be the silent driver of rapid deterioration. Ultimately, the integration of SHAP-based individual explanations facilitates a transition from generalized protocols to precision pediatric care, enabling earlier and more targeted interventions based on the unique physiological state of each child.

### 4.1 Research limitations and future prospects

This study has several limitations that warrant detailed consideration. First, as a single-center retrospective study conducted at the First Affiliated Hospital of Henan University of Medical Sciences, the model’s decision-making logic may be influenced by local clinical protocols. Crucially, while our tertiary center routinely performs flow cytometry and cytokine panels within 24 h of admission, we recognize that key predictive features like T-lymphocyte subsets and IL-6 are not routinely available in primary or secondary pediatric care settings. Consequently, the stated goal of a “real-time EMR-based early warning” may not be universally feasible. In clinical practice, our model is best positioned either as a specialized assessment tool for equipped tertiary centers or as a “step-up” diagnostic panel triggered when patients present with primary red flags (such as dyspnea and isolated high CRP) but require deeper immunological profiling to guide advanced therapies. To facilitate this clinical translation, we propose a simplified Clinical Decision Support (CDS) dashboard layout for EMR embedding. The interface would consist of three core modules: (1) A “Traffic-Light” Risk Gauge displaying the real-time probability of severe illness (Green: Low, Yellow: Intermediate, Red: High); (2) A dynamic “Top 3 Risk Drivers” list (derived directly from the patient’s local SHAP values) alerting the clinician to the specific physiological factors driving the risk (e.g., “Warning: Critical drop in CD3+T-cells combined with high CRP”); and (3) A “Clinical Action Suggestion” module linking the identified risk strata to institutional treatment pathways (e.g., prompting consideration for intensified monitoring or early immunomodulatory therapy).

Second, although 402 participants were included, the number of severe cases (*n* = 125) remains relatively small for high-capacity machine learning. The significant disparity between the training set AUC (0.988) and the held-out internal test set AUC (0.805) is a clear indicator of overfitting, suggesting that the Gradient Boosting Machine (GBM) may have partially “memorized” noise specific to our small cohort rather than learning purely universal pathological patterns. To explicitly and quantitatively mitigate this inherent risk, we prioritized a GBM model with internal regularization (optimized via grid search with 10-fold cross-validation), and strictly compressed the initial 32 candidate variables down to an optimal set of 16 core features, which was crucial for preserving statistical power, reducing noise, and enhancing the stability of the final model.

Third, the requirement for complete clinical and immunological records led to the exclusion of 699 patients. This “complete case” approach introduces a notable selection bias; the model was essentially validated on a “filtered” population of children who remained stable enough to complete a full diagnostic workup within 24 h. This likely leads to an over-optimistic estimation of model performance compared to a real-world clinical environment where missing data and rapid clinical deterioration are common. However, our newly added multiple imputation-based sensitivity analysis on the full eligible cohort (*n* = 1,101) maintained an AUC of 0.887, mitigating concerns that the identified clinical-immunological signatures were merely artifacts of the complete-case subpopulation. Nevertheless, while our model demonstrated acceptable internal validity across specific age subgroups (AUC 0.833 for <5 years; AUC 0.776 for ≥5 years), establishing its broad generalizability is paramount. The external validity of these age-specific performance metrics and the universal applicability of our identified immunological thresholds remain limited by our single-center design. Therefore, future multi-center, prospective cohorts with true external validation are strictly required to refine the model’s robustness and ensure its clinical reliability across diverse, unselected, and geographically distinct patient populations.

Traditional predictive models for severe pneumonia are largely based on Logistic Regression, which operates on the core assumption of a linear additive relationship between predictors and risk. However, by utilizing the GBM algorithm and SHAP dependency plots, this study uncovers the complex non-linear characteristics and threshold effects inherent in SMPP progression, marking a fundamental departure from previous research.

First, SHAP dependency plots reveal that key indicators do not drive risk at a constant, uniform rate; instead, they exhibit distinct “mutation thresholds.” For instance, when C-reactive protein (CRP) exceeds a specific level, or the percentage of total T lymphocytes (CD3+) falls below a critical tipping point, the risk of severe illness surges exponentially rather than linearly. This “all-or-nothing” threshold effect is highly valuable in clinical practice. It alerts physicians that once a biomarker crosses a specific “red line,” the patient’s condition may undergo a qualitative shift—a critical diagnostic detail that traditional linear models often “smooth over.”

Second, the most significant finding of this study is the synergistic interaction effect between features. SHAP interaction analysis reveals a “double-hit” mechanism of inflammation and immunity: in the context of high CRP levels, the positive contribution of low CD3+counts to the total risk is significantly amplified. In traditional linear regression, such relationships require researchers to pre-define and manually introduce interaction terms, which are mathematically ill-equipped to handle the complex superposition of high-dimensional features. Conversely, the gradient boosting algorithm automatically identifies these “1 + 1 > 2” non-linear patterns, capturing the acute risk surge when dyspnea, high inflammatory load, and cellular immune exhaustion coexist.

This deep exploration of biological complexity demonstrates the true value of machine learning in SMPP risk stratification. It does not merely improve predictive precision (AUC 0.805); more importantly, it identifies non-linear, threshold-based clinical signatures that provide clinicians with a digital decision-making tool far more aligned with the actual dynamic patterns of disease evolution.

## 5 Conclusion

In summary, this study successfully constructed an early warning model for severe MPP (severe *Mycoplasma pneumoniae* pneumonia) risk in children based on the GBM algorithm, demonstrating good predictive efficacy. More importantly, through SHAP interpretability analysis, it not only validated the core status of traditional severe illness indicators such as dyspnea and CRP, but also revealed the significant contribution of host immune status (total T lymphocytes) and the synergistic worsening effect among different risk factors. This model combines data-driven predictive capabilities with clinically understandable decision-making support, providing clinicians in equipped tertiary settings with a powerful tool for early identification of high-risk children and implementation of stratified management. However, as this is currently a single-center model, prospective multi-center validation is strictly required before it can be integrated into electronic medical record (EMR) systems for widespread clinical application.

## Data Availability

The original contributions presented in the study are included in the article/Supplementary material, further inquiries can be directed to the corresponding authors.

## Supplementary material

SUPPLEMENTARY FIGURE 1 Research flowchart.

SUPPLEMENTARY FIGURE 2 Calibration plot of the GBM model on the held-out internal test set.

SUPPLEMENTARY FIGURE 3 Individualized risk prediction using SHAP force plots for representative intermediate-risk and low-risk cases. The SHAP force plots illustrate the model's decision-making process for (A) an intermediate-risk child and (B) a low-risk child. The horizontal axis represents the predicted risk probability of severe illness. The value E[f(x)] = 0.26 indicates the model's baseline average risk prediction for the cohort. The solid line and the value f(x) represent the model's final risk prediction for each specific patient. Red-shaded features (positive SHAP values) drive the prediction towards a higher risk of severity, while yellow/light-shaded features (negative SHAP values) act as protective factors, pushing the risk toward lower values.
