# Supplementary material for: Machine learning-driven risk assessment of severe Mycoplasma pneumoniae in children: analysis based on core clinical and immunological features
Source: Front Med (Lausanne). 2026 Jul 21;13:1843690. doi: 10.3389/fmed.2026.1843690 (PMC13433356; doi:10.3389/fmed.2026.1843690)
Supplement: Supplementary file 1 [file Table_1.DOCX]

**Supplementary Table 1: Basic Characteristics and Difference Analysis**

| Variables | Total (n = 402) | NSMPP (n = 277) | SMPP (n = 125) | Statistic | *P* |
| --- | --- | --- | --- | --- | --- |
|  |  |  |  |  |  |
| Age, M (Q₁, Q₃) | 6.13 (4.00, 8.00) | 6.18 (4.00, 8.00) | 6.00 (3.67, 8.00) | Z=-0.84 | 0.400 |
| Medical history, M (Q₁, Q₃) | 8.00 (5.00, 12.00) | 7.00 (5.00, 12.00) | 9.00 (5.00, 13.00) | Z=-1.01 | 0.311 |
| Cough, M (Q₁, Q₃) | 7.00 (4.00, 10.00) | 7.00 (4.00, 11.00) | 7.00 (3.00, 10.00) | Z=-0.50 | 0.620 |
| Fever, M (Q₁, Q₃) | 7.00 (5.00, 11.00) | 7.00 (5.00, 10.00) | 9.00 (6.00, 12.00) | Z=-1.74 | 0.082 |
| WBC, M (Q₁, Q₃) | 8.55 (6.58, 11.75) | 8.48 (6.40, 11.78) | 8.76 (6.90, 11.67) | Z=-0.99 | 0.325 |
| CRP, M (Q₁, Q₃) | 21.09 (7.45, 45.00) | 17.00 (5.63, 37.20) | 34.67 (11.70, 95.56) | Z=-5.28 | <.001 |
| PCT, M (Q₁, Q₃) | 0.28 (0.00, 1.00) | 0.22 (0.00, 0.93) | 0.41 (0.03, 1.23) | Z=-3.48 | <.001 |
| LDH, M (Q₁, Q₃) | 398.00 (271.50, 643.50) | 358.00 (261.00, 603.00) | 449.00 (299.00, 698.00) | Z=-2.65 | 0.008 |
| IL-6, M (Q₁, Q₃) | 19.80 (7.21, 47.81) | 16.05 (6.82, 41.78) | 26.30 (9.94, 69.00) | Z=-2.97 | 0.003 |
| D2D, M (Q₁, Q₃) | 1.40 (0.90, 4.00) | 1.00 (0.79, 3.00) | 2.50 (1.20, 5.60) | Z=-5.70 | <.001 |
| Erythrocyte sedimentation rate, M (Q₁, Q₃) | 25.00 (17.00, 37.75) | 25.00 (16.00, 38.00) | 25.00 (17.00, 37.00) | Z=-0.18 | 0.855 |
| ALT, M (Q₁, Q₃) | 17.00 (12.00, 26.00) | 17.00 (12.00, 26.00) | 19.00 (13.00, 34.00) | Z=-2.00 | 0.046 |
| AST, M (Q₁, Q₃) | 28.00 (22.00, 42.00) | 27.00 (22.00, 39.00) | 32.00 (24.00, 51.00) | Z=-3.11 | 0.002 |
| CK, M (Q₁, Q₃) | 60.00 (37.00, 116.50) | 60.00 (35.00, 106.00) | 66.00 (41.00, 139.00) | Z=-1.96 | 0.049 |
| CKMB, M (Q₁, Q₃) | 2.03 (1.30, 3.10) | 1.97 (1.30, 3.00) | 2.09 (1.30, 3.20) | Z=-0.73 | 0.462 |
| MP sequence number, M (Q₁, Q₃) | 12300000.00 (114000.00, 119750000.00) | 1,320,000.00 (126,000.00, 93,000,000.00) | 8670000.00 (110000.00, 197000000.00) | Z=-0.35 | 0.727 |
| Immunoglobulin G , g/L, M (Q₁, Q₃) | 7.75 (4.90, 10.70) | 7.60 (5.10, 10.70) | 7.90 (4.40, 10.30) | Z=-0.19 | 0.847 |
| Immunoglobulin A , g/L, M (Q₁, Q₃) | 1.00 (0.44, 1.55) | 1.00 (0.38, 1.51) | 1.04 (0.59, 1.69) | Z=-2.08 | 0.038 |
| Immunoglobulin M , g/L, M (Q₁, Q₃) | 0.99 (0.60, 1.40) | 1.00 (0.62, 1.41) | 0.97 (0.53, 1.38) | Z=-0.72 | 0.474 |
| Complement C3 , g/L, M (Q₁, Q₃) | 1.05 (0.88, 1.29) | 1.07 (0.88, 1.29) | 1.03 (0.89, 1.25) | Z=-0.48 | 0.634 |
| Complement C4 , g/L, M (Q₁, Q₃) | 0.26 (0.18, 0.35) | 0.27 (0.18, 0.35) | 0.25 (0.18, 0.35) | Z=-0.39 | 0.695 |
| Total T lymphocytes ( %), M (Q₁, Q₃) | 0.54 (0.45, 0.64) | 0.55 (0.45, 0.64) | 0.52 (0.45, 0.61) | Z=-1.60 | 0.110 |
| CD3+CD8+ , (%), M (Q₁, Q₃) | 0.20 (0.16, 0.25) | 0.20 (0.16, 0.25) | 0.20 (0.16, 0.25) | Z=-0.19 | 0.853 |
| CD3+CD4+ , (%), M (Q₁, Q₃) | 0.32 (0.24, 0.40) | 0.32 (0.24, 0.40) | 0.31 (0.25, 0.39) | Z=-0.94 | 0.345 |
| CD3+CD4+CD8- , (%), M (Q₁, Q₃) | 0.05 (0.03, 0.06) | 0.05 (0.03, 0.06) | 0.05 (0.04, 0.06) | Z=-2.32 | 0.021 |
| CD3+CD4+/CD3+CD8+, (%) , M (Q₁, Q₃) | 0.45 (0.02, 0.58) | 0.45 (0.02, 0.56) | 0.47 (0.03, 0.60) | Z=-1.64 | 0.102 |
| CD19+ B lymphocytes , (%), M (Q₁, Q₃) | 0.23 (0.19, 0.25) | 0.23 (0.19, 0.25) | 0.23 (0.20, 0.26) | Z=-0.30 | 0.762 |
| CD56+ NK cells , (%), M (Q₁, Q₃) | 0.20 (0.08, 0.32) | 0.20 (0.07, 0.31) | 0.23 (0.08, 0.33) | Z=-1.56 | 0.118 |
| CD3+CD56+ T-NK cells , (%), M (Q₁, Q₃) | 0.03 (0.01, 0.07) | 0.04 (0.01, 0.07) | 0.03 (0.01, 0.07) | Z=-0.82 | 0.410 |
| Gender, n(%) |  |  |  | χ²=1.85 | 0.174 |
| female | 192 (47.76) | 126 (45.49) | 66 (52.80) |  |  |
| male | 210 (52.24) | 151 (54.51) | 59 (47.20) |  |  |
| Difficulty breathing, n(%) |  |  |  | χ²=65.94 | <.001 |
| no | 347 (86.32) | 265 (95.67) | 82 (65.60) |  |  |
| yes | 55 (13.68) | 12 (4.33) | 43 (34.40) |  |  |
| Sputum plug shaping, n(%) |  |  |  | χ²=23.00 | <.001 |
| no | 267 (66.42) | 205 (74.01) | 62 (49.60) |  |  |
| yes | 135 (33.58) | 72 (25.99) | 63 (50.40) |  |  |
| Pleural effusion, n(%) |  |  |  | χ²=12.19 | <.001 |
| no | 291 (72.39) | 215 (77.62) | 76 (60.80) |  |  |
| yes | 111 (27.61) | 62 (22.38) | 49 (39.20) |  |  |
| Complications, n(%) |  |  |  | χ²=3.03 | 0.082 |
| no | 275 (68.41) | 197 (71.12) | 78 (62.40) |  |  |
| yes | 127 (31.59) | 80 (28.88) | 47 (37.60) |  |  |
| Cardiovascular injury , n(%) |  |  |  | χ²=22.26 | <.001 |
| no | 361 (89.80) | 262 (94.58) | 99 (79.20) |  |  |
| yes | 41 (10.20) | 15 (5.42) | 26 (20.80) |  |  |
| Liver injury , n(%) |  |  |  | χ²=0.46 | 0.496 |
| no | 366 (91.04) | 254 (91.70) | 112 (89.60) |  |  |
| yes | 36 (8.96) | 23 (8.30) | 13 (10.40) |  |  |
| Anemia, n(%) |  |  |  | χ²=11.85 | <.001 |
| no | 362 (90.05) | 259 (93.50) | 103 (82.40) |  |  |
| yes | 40 (9.95) | 18 (6.50) | 22 (17.60) |  |  |
| Urinary tract injury , n(%) |  |  |  | χ²=4.31 | 0.038 |
| no | 373 (92.79) | 262 (94.58) | 111 (88.80) |  |  |
| yes | 29 (7.21) | 15 (5.42) | 14 (11.20) |  |  |
| Systemic inflammatory response syndrome, n(%) |  |  |  | χ²=9.53 | 0.002 |
| no | 331 (82.34) | 239 (86.28) | 92 (73.60) |  |  |
| yes | 71 (17.66) | 38 (13.72) | 33 (26.40) |  |  |
| Coagulation dysfunction , n(%) |  |  |  | χ²=25.30 | <.001 |
| no | 365 (90.80) | 265 (95.67) | 100 (80.00) |  |  |
| yes | 37 (9.20) | 12 (4.33) | 25 (20.00) |  |  |
| Z: Mann-Whitney test, χ²: Chi-square test | | | | | |
| M: Median, Q₁: 1st Quartile, Q₃: 3st Quartile | | | | | |

**Supplementary Table 2: Categorized List of Evaluated Machine Learning Algorithms and Ensemble Variants**

| Base Model Family | Specific Algorithms & Ensemble Variants Included |
| --- | --- |
| Tree-Based Models & Variants | Single Models: GBM, RF, XGBoost.  Ensembles containing Tree Models: glmBoost+GBM, Stepglm[both]+GBM, Stepglm[backward]+GBM, Lasso+GBM, RF+GBM, glmBoost+RF, Stepglm[both]+RF, Lasso+RF, Stepglm[backward]+RF, glmBoost+XGBoost, Lasso+XGBoost, RF+XGBoost, Stepglm[both]+XGBoost, Stepglm[backward]+XGBoost, RF+SVM, RF+Ridge, RF+Enet[alpha=0.1~0.9], RF+plsRglm, RF+Stepglm[forward], RF+Lasso, RF+glmBoost, RF+Stepglm[both], RF+Stepglm[backward], RF+LDA, RF+NaiveBayes. |
| Linear & Penalized Regressions | Single Models: glmBoost, Lasso, Ridge, Enet[alpha=0.1~0.9], Stepglm[forward], Stepglm[both], Stepglm[backward], plsRglm.  Ensembles of Linear Models: Lasso+Stepglm[both], glmBoost+Ridge, glmBoost+Enet[alpha=0.1~0.9], Lasso+glmBoost, Lasso+plsRglm, glmBoost+plsRglm, glmBoost+Stepglm[forward], Lasso+Stepglm[forward], Stepglm[both]+Ridge, Stepglm[backward]+Ridge, Stepglm[both]+plsRglm, Stepglm[backward]+plsRglm, Stepglm[both]+Enet[alpha=0.1~0.9], Stepglm[backward]+Enet[alpha=0.1~0.9], Stepglm[both]+Lasso, Stepglm[backward]+Lasso, Lasso+Stepglm[backward], glmBoost+Stepglm[both], glmBoost+Stepglm[backward], Stepglm[both]+glmBoost, Stepglm[backward]+glmBoost. |
| Support Vector Machines (SVM) | Single Model: SVM.  Ensembles containing SVM: Lasso+SVM, glmBoost+SVM, Stepglm[both]+SVM, Stepglm[backward]+SVM. (Note: RF+SVM is listed under Tree-Based Models). |
| Other Approaches | Single Models: LDA, NaiveBayes.  Ensembles containing LDA or NaiveBayes: glmBoost+LDA, Stepglm[both]+LDA, Stepglm[backward]+LDA, Lasso+LDA, Lasso+NaiveBayes, glmBoost+NaiveBayes, Stepglm[both]+NaiveBayes, Stepglm[backward]+NaiveBayes. (Note: RF+LDA and RF+NaiveBayes are listed under Tree-Based Models). |

**Supplementary Table 3: Subgroup Analysis of GBM Model Performance on the Held-out Internal Test Set**

| Subgroup | Total Patients (n) | Severe Cases (n) | AUC (95% CI) |
| --- | --- | --- | --- |
| Age |  |  |  |
| < 5 years | 40 | 10 | 0.833 (0.638–0.981) |
| ≥ 5 years | 80 | 26 | 0.776 (0.640–0.884) |
| Sex |  |  |  |
| Male | 64 | 17 | 0.761 (0.605–0.890) |
| Female | 56 | 19 | 0.791 (0.643–0.916) |
